# Supplementary material for: Assessment and Training of Perceptual-Motor Function: Performance of College Wrestlers Associated with History of Concussion
Source: Brain Sci. 2024 Jan 10;14(1):68. doi: 10.3390/brainsci14010068 (PMC10813796; doi:10.3390/brainsci14010068)
Supplement: Supplementary file 1 [file brainsci-14-00068-s001.zip › Table S1.pdf]

Table S1. Pre- and Post-Training Mean ± SD among Athletes Reporting No Concussion (NoC) versus History of Concussion (HxC) and Shapiro-Wilk test for distribution normality *p*-value (SW-*p*) for Perceptual Latency Intra-Individual Variability (PL-IIV), Response Time Intra-Individual Variability (RT-IIV), and 40-Trial Perceptual Latency Average (PL-Avg).

		Pre-Training			Post-Training		
Metric	Group	Mean ± SD	S-W *p*	S-W *p**	Mean ± SD	S-W *p*	S-W *p**
Neck PL-IIV	NoC<br>HxC	0.211 ± 0.106<br>0.382 ± 0.122	0.018	0.994	0.143 ± 0.057<br>0.442 ± 0.614	<0.001	0.088
Arm PL-IIV	NoC<br>HxC	0.226 ± 0.135<br>0.321 ± 0.169	<0.001	0.548	0.139 ± 0.045<br>0.253 ± 0.117	<0.001	0.572
Step PL-IIV	NoC<br>HxC	0.235 ± 0.151<br>0.364 ± 0.247	<0.001	0.046	0.160 ± 0.054<br>0.277 ± 0.123	0.001	0.338
Neck RT-IIV	NoC<br>HxC	0.233 ± 0.080<br>0.310 ± 0.087	0.109	0.217	0.167 ± 0.073<br>0.302 ± 0.167	0.002	0.453
Arm RT-IIV	NoC<br>HxC	0.230 ± 0.107<br>0.295 ± 0.081	0.018	0.467	0.143 ± 0.049<br>0.267 ± 0.121	<0.001	0.097
Step RT-IIV	NoC<br>HxC	0.234 ± 0.104<br>0.314 ± 0.087	0.004	0.072	0.168 ± 0.074<br>0.266 ± 0.111	0.011	0.204
Neck PL-Avg	NoC<br>HxC	0.617 ± 0.104<br>0.639 ± 0.095	0.484	0.994	0.570 ± 0.061<br>0.672 ± 0.151	0.003	0.088
Arm PL-Avg	NoC<br>HxC	0.727 ± 0.120<br>0.769 ± 0.125	0.213	0.548	0.609 ± 0.083<br>0.646 ± 0.077	0.618	0.572
Step PL-Avg	NoC<br>HxC	0.718 ± 0.127<br>0.759 ± 0.124	0.004	0.046	0.617 ± 0.072<br>0.675 ± 0.081	0.650	0.338

* Shapiro-Wilk *p*-value after Log_e transformation (increased value = decreased deviation from normality)
